# Dosage effect on uropathogenic *Escherichia coli *anti-adhesion activity in urine following consumption of cranberry powder standardized for proanthocyanidin content: a multicentric randomized double blind study

**DOI:** 10.1186/1471-2334-10-94

**Published:** 2010-04-14

**Authors:** Amy B Howell, Henry Botto, Christophe Combescure, Anne-Béatrice Blanc-Potard, Lluis Gausa, Tetsuro Matsumoto, Peter Tenke, Albert Sotto, Jean-Philippe Lavigne

**Affiliations:** 1 Marucci Center for Blueberry and Cranberry Research and Extension, Rutgers, The State University of New Jersey, Chatsworth, NJ, USA; 2 Service d'Urologie, Hôpital Foch, Suresnes, France; 3 Faculté de Médecine, Université de Genève, Genève, Switzerland; 4 Institut National de la Santé et de la Recherche Médicale, Espri26, Université Montpellier 1, Nîmes, France; 5 Fundació Puigvert, Barcelona, Spain; 6 Department of Urology, Graduate School of Medical Sciences, Kyushu University, Kitakyushu, Japan; 7 Department of Urology, South-Pest Hospital, Budapest, Hungary; 8 Laboratoire de Bactériologie, Virologie, Parasitologie, University Hospital Carémeau, Nîmes, France

## Abstract

**Background:**

Ingestion of cranberry (*Vaccinium macrocarpon *Ait.) has traditionally been utilized for prevention of urinary tract infections. The proanthocyanidins (PACs) in cranberry, in particular the A-type linkages have been implicated as important inhibitors of primarily P-fimbriated *E. coli *adhesion to uroepithelial cells. Additional experiments were required to investigate the persistence in urine samples over a broader time period, to determine the most effective dose per day and to determine if the urinary anti-adhesion effect following cranberry is detected within volunteers of different origins.

**Methods:**

Two separate bioassays (a mannose-resistant hemagglutination assay and an original new human T24 epithelial cell-line assay) have assessed the ex-vivo urinary bacterial anti-adhesion activity on urines samples collected from 32 volunteers from Japan, Hungary, Spain and France in a randomized, double-blind versus placebo study. An *in vivo Caenorhabditis elegans *model was used to evaluate the influence of cranberry regimen on the virulence of *E. coli *strain.

**Results:**

The results indicated a significant bacterial anti-adhesion activity in urine samples collected from volunteers that consumed cranberry powder compared to placebo (p < 0.001). This inhibition was clearly dose-dependent, prolonged (until 24 h with 72 mg of PAC) and increasing with the amount of PAC equivalents consumed in each cranberry powder regimen. An *in vivo Caenorhabditis elegans *model showed that cranberry acted against bacterial virulence: *E. coli *strain presented a reduced ability to kill worms after a growth in urines samples of patients who took cranberry capsules. This effect is particularly important with the regimen of 72 mg of PAC.

**Conclusions:**

Administration of PAC-standardized cranberry powder at dosages containing 72 mg of PAC per day may offer some protection against bacterial adhesion and virulence in the urinary tract. This effect may offer a nyctohemeral protection.

## Background

Ingestion of cranberry (*Vaccinium macrocarpon *Ait.) has traditionally been utilized for prevention of urinary tract infections (UTI). A recent systematic review concluded that there is some positive clinical evidence that consumption of cranberry juice can reduce the number of symptomatic UTIs in women over a 12-month period [1]. Research suggests that consuming cranberry products may prevent adhesion of certain *Escherichia coli *strains to the uroepithelium [2-4], interfering with this important initial step in the infection process [5]. The proanthocyanidins (PACs) in cranberry, in particular the A-type linkages have been implicated as important inhibitors of primarily P-fimbriated *E. coli *adhesion to uroepithelial cells *in vitro *[6-9] and *ex vivo *[10]. Cranberry PAC extracts inhibited adhesion of *E. coli *in a linear, dose-dependent fashion over a PAC concentration range of 75 to 5 μg/mL [9]. The PACs in cranberry contain unusual double A-type linkages which may be important structural features in the anti-adhesion process [7]. In one study, other food sources of PAC that contain only B-type linkages (chocolate, grape, apple and green tea) were consumed. However, they did not elicit *ex vivo *bacterial anti-adhesion activity. Only cranberry juice with A-type PACs prevented bacterial adhesion [11].

Several assays have been successfully utilized to detect bacterial anti-adhesion activity of cranberry, including mannose-resistant hemagglutination (MRHA) [3,7,9,12], Gal-Gal receptor bead agglutination [3,6], bladder epithelial cell adherence [2,3,9,12,13] and microplate-turbidity [14]. Utilizing a bioassay to detect *in vitro *anti-adhesion activity of whole cranberry products and isolated PACs is useful for determining product integrity, however it does not assess *in vivo *activity of the post-ingested cranberry metabolites. Urinary bacterial anti-adhesion activity may be a more biologically relevant marker for cranberry ingestion as well as the effectiveness of consuming cranberry for prevention of UTI. A number of studies have demonstrated an *ex vivo *bacterial anti-adhesion effect in human urine following consumption of different cranberry products [11-13,15-17].

The current recommended daily dosage of cranberry for UTI prevention is based on the efficacious levels that have been administered in human intervention trials. A commonly recommended amount of cranberry for UTI prevention is daily consumption of 300 mL of cranberry juice cocktail containing 36 mg proanthocyanidins measured by DMAC method, which clinically reduced bacteriuria and pyuria [18]. Formulations of cranberry in powder form like tablets and capsules have demonstrated activity *in vitro *[9], *ex vivo *[19] and *in vivo *[20,21]. However, additional human studies are needed to more comprehensibly establish the effective dose response range for cranberry, utilizing products standardized for PAC content. Standardization is important to establish product integrity and shelf-life, and to formulate accurate test materials for use in research studies. Criticism of early studies on cranberry often cited lack of use of appropriate standardized material, making conclusions and comparisons to other studies difficult [1]. Research indicates that processing of cranberry into various products can impact the PAC composition, which is very heterogeneous [22]. Molecular weight and location of the A-type linkages in cranberry PACs could potentially impact the bacterial anti-adhesion activity [23]. Thus, proper standardization of cranberry products for PAC content and correlation of the PAC level with anti-adhesion bioactivity may be important to ensure that particular cranberry products contain PACs that are efficacious.

A first double-blind, randomized, placebo-controlled study of our team indicated a dose-dependent effect in *ex vivo *urinary bacterial anti-adhesion activity after consumption of a PAC-standardized commercial cranberry powder [12]. Anti-adhesion activity was measured in urines 12 h after treatment regimes. Following these results, additional experiments were required to investigate the persistence in urine over a broader time period, and to determine the most effective dose of PAC equivalents per day. Further studies are also needed to determine if the urinary anti-adhesion effect following cranberry is universal within the population or is specific to certain ethnicities, dietary regimes, locations, etc. To address these issues, we conducted the present multi-location randomized, double-blind versus placebo study to evaluate additional dosage regimes and collection time-periods following ingestion of the same PAC-standardized cranberry powder.

## Methods

Even if cranberry is a supplement food, the study has been approved by the different research ethics committees (1/Comité d'éthique Sud Méditerranée III, Nîmes, France; 2/Comité Etico de Investigaciòn Clinica (CEIC) de Fundació Puigvert, Barcelona, Spain; 3/Ethical committee of Jahn Ferenc South-Pest Teaching Hospital, Hungary; 4/Ethics Committee of Medicine and Medical Care, University of Occupational and Environmental Health, Japan), in each country and has been conducted according to the principles expressed in the Declaration of Helsinki. Thirty-two female, sexually-active volunteers over the age of 18, with normal renal function were recruited through four urology departments in Japan (Kyushu University Hospital), Hungary (South-Pest Hospital, Budapest), Spain (Fundació Puigvert, Barcelona), and France (Hôpital Foch, Suresnes) (eight volunteers/department) to participate in this randomized, double-blind, placebo-controlled cross-over study. Volunteers were cleared through ethics committees and provided informed consent. Exclusion criteria included antibiotic use within six months prior to the study, pregnancy, known allergy or intolerance of cranberry products, or routine consumption of any food supplements consisting of vitamins, minerals or trace elements. Throughout the study, volunteers were instructed not to alter their dietary or lifestyle habits in any way. However, during the capsule consumption, volunteers were told to avoid all *Vaccinium*-containing foods, drinks and supplements including other forms of *V. macrocarpon *(cranberry), *V. myrtillus *(bilberry or European blueberry), *V. angustifolium *(wild or lowbush blueberry), *V. corymbosum *(highbush blueberry), and *V. vitis-ideae *(lingonberry). In addition, volunteers were instructed to limit consumption of chocolate, tea, grape and wine. All medications taken during the study were reported to the doctor responsible for following the study at each center.

The study was carried out using commercially available capsules of cranberry powder (Urell^®^/Ellura™ Pharmatoka, Rueil Malmaison, France) and capsules of placebo composed of colloidal silica, magnesium stearate, cellulose and gelatin. Capsules were opaque to conceal the color of the contents. The PACs in the cranberry powder were quantified by Brunswick Laboratories (Norton, MA) using a colorimetric assay, an updated dimethylaminocinnamaldehyde method (DMAC method), taking advantage of the selective colorimetric reaction between PACs and DMAC after open column gel chromatography on Sephadex^® ^LH-20 (Amersham). The capsule dosages were standardized to deliver 18 or 36 mg of PAC equivalents in the cranberry powder.

The volunteers in Japan and Hungary received 0, 36 or 72 mg PAC equivalents per day and those in France and Spain received 0, 18 or 36 mg PAC equivalents per day. Each volunteer received successively three regimens (always 2 capsules) distributed in random order, consisting of: (1) cranberry (2 PAC dosage levels), or (2) placebo, or (3) 1 capsule of cranberry at each PAC dosage and 1 capsule of placebo, with a washout period of at least one week between each regimen. Volunteers consumed the capsules in the morning at 8:00 AM. The first urines were collected from 9:00 AM-2:00 PM following capsule consumption, and pooled. The second collections were made the following morning (8:00 AM). Pre-cranberry consumption urines collections (0 h) were obtained at the beginning of the study in each volunteer. Various biological and physicochemical parameters of the urine samples (collected at each regimen) were measured using the Multistix^® ^(Bayer) system. Urines with an abundance of leukocytes and nitrites were excluded. Remaining samples were centrifuged at 4000 g for 15 minutes, sterilized by filtration (0.45 μm), separated in 3 aliquots and stored at -20°C.

A uropathogenic *E. coli *strain previously isolated from a patient with UTI (NECS978323) [12], with P-fimbriae *papG *and type-1 pili was utilized. To allow the direct observation of adherent bacteria by fluorescence microscopy, NECS978323 was genetically modified to express green fluorescent protein (GFP) using a pBBR-derived non-mobilizable plasmid carrying a GFP expression cassette [24]. Bacteria were grown in trypticase soy broth (bioMerieux, Marnes La Coquette, France) or colonization factor antigen agar for 16 h at 37°C to enhance expression of P-fimbriae.

To insure product potency, the PAC-standardized cranberry powder was tested for *in vitro *uropathogenic bacterial anti-adhesion activity prior to consumption by the volunteers, utilizing a MRHA, to detect anti-adhesion activity in uropathogenic P-fimbriated *E. coli *[11]. Briefly, the anti-adhesion bioactivity of the powder was tested by measuring the ability of the fractions to suppress agglutination of human red blood cells (HRBC) (A1, Rh+) following incubation with uropathogenic P-fimbriated *E. coli*. Bacteria were suspended in phosphate-buffered saline (PBS) solution at pH 7.0 at a concentration of 5 × 10^8 ^bacteria/mL. The powder was dissolved in PBS at a starting concentration of 60 mg/mL, and the pH adjusted to neutrality with NaOH. A serial 2-fold dilution was prepared, and each dilution (30 mL) was incubated with 10 mL of bacterial suspension on a 24-well polystyrene plate for 10 min at room temperature on a rotary shaker. A 3% v/v suspension of HRBCs in PBS was prepared, and 10 mL of the diluted blood was added to the test suspension. The suspension was incubated for 20 min on a rotary shaker at 21C and evaluated microscopically for the ability to prevent agglutination. The final dilution concentration was recorded at which agglutination suppression by the cranberry fraction occurred. Wells containing bacteria plus PBS, HRBC plus PBS, bacteria plus test fraction, and HRBC plus test fraction served as negative controls for agglutination, and wells containing bacteria plus HRBC served as a positive control for agglutination. Assays were performed in triplicates.

Urines were tested *ex vivo *for anti-adhesion activity before and after the treatment regimes, utilizing two separate assays. The MRHA assay described above was modified by substituting urine for a cranberry solution. Briefly, a 30-μL drop of each urine was incubated with 10 μL of the bacterial suspension on a 24-well polystyrene plate for 10 min at 21C on a rotary shaker. The HRBCs were added to the urines, incubated for 20 min on a rotary shaker at 21C and evaluated microscopically for the ability to prevent agglutination. If no agglutination was observed, the urine was considered to contain anti-adhesion metabolites and was recorded as possessing Anti-Adhesion Activity (AAA). The results were expressed as a percentage of anti-adhesion activity (0% MRHA = 100% AAA, 50% MRHA = 50% AAA and 100% MRHA = 0% AAA). Assays were repeated 4 times on triplicate urine samples and the standard error calculated.

In the second *ex vivo *urine assay, bacterial adhesion was evaluated utilizing the human T24 epithelial cell-line (ATCC HTB-4). A new technology was developed using fluorescent NECS978323 to enhance detection of strain adhesion. Monolayers of epithelial cells were grown at 37°C in McCoy's 5a medium containing 10% (v/v) fetal calf serum, 1.5 mM glutamine, and antibiotics (50 U/mL penicillin and 50 mg/mL of streptomycin), on coverslips in 24-well Falcon tissue culture plates. Bacteria were grown overnight in the urine samples containing 5% (v/v) Luria broth. Bacterial were harvested by centrifugation, resuspended at DO_600 _0.1 in McCoy's medium, added to the tissue culture and incubated for 2.5 h at 37°C. After washes with PBS, the cells were fixed with 4% paraformaldehyde, incubated 20 min at room temperature, and examined under a fluorescent microscope. An adhesion index (AI) corresponding to the mean number of adherent bacteria per cell for 100 cells was calculated. This index was expressed as the mean of at least three independent assays.

*C. elegans *has been used to develop an easy model system of host-pathogen interactions to identify basic evolutionarily conserved pathways associated with microbial pathogenesis. This test is based upon the capacity of *E. coli *to be ingested by *C. elegans *nematodes leading to infection and ultimately involving the killing death of the worms [25]. Percentage of killed nematodes in presence of the *E. coli *strain is an indirect marker of virulence potential of this strain. The *C. elegans *infection assay was carried out as described by Lavigne et al. [25] using Fer-15 mutant line, which has a temperature sensitive fertility defect. Fer-15 was provided by the *Caenorhabditis *Genetics Center, which is funded by the NIH National Center for Research Resources (NCRR). Briefly to synchronize the growth of worms, eggs were collected using the hypochlorite method. *E. coli *strain was grown for overnight in human urine containing 5% (v/v) nematode growth medium (NGM). Bacteria cells were harvested by centrifugation, washed once and suspended in phosphate buffered saline solution (PBS) at pH 7.0 at a concentration of 10^5 ^CFU/ml. NGM agar plates were inoculated with 10 μl of strain and incubated at 37°C for 8-10 h. Plates were allowed to cool to room temperature and seeded with L4 stage worms (20-30 per plate). Plates were then incubated at 25°C and scored each day for live worms under a stereomicroscope (Leica MS5). At least three replicates repeated 5 times were performed for each selected clone. A worm was considered dead when it no longer responded to touch. Worms that died as a result of becoming stuck to the wall of the plate were excluded from the analysis. Lethal Time 50% (LT50) and death (LT100) corresponded to time (in hours) required to kill 50% and 100% of the nematode population, respectively. OP50 is an avirulent *E. coli *strain, an international standard food for nematodes used as control. The number of bacteria within the *C. elegans *digestive tract was carried out as described by Garsin et al. [26]. Five *C. elegans *were picked at 72 h, and the surface bacteria were removed by washing the worms twice in 4 μl drops of M9 medium on a NGM agar plate containing 25 μg/ml gentamicin. The nematodes were placed in a 1.5 ml Eppendorf tube containing 20 μl of M9 medium with 1% Triton X-100 and were mechanically disrupted by using a pestle. The volume was adjusted to 50 μl with M9 medium containing 1% Triton X-100 which was diluted and plated on Luria-Bertani agar containing 50 μg/ml ampicillin. At least three replicates were performed for each assay.

The quantitative variables were described by the median values, the range and the mean, and standard deviation. The qualitative variables were described by figures and percentages. The 95% confidence intervals were assessed by the exact method of Clopper-Pearson. Frequencies between AAA = 0% and AAA > 0% were compared according to the criteria using a Fisher exact test and the index values were compared using a Kruskal-Wallis test. The index value was modelled by the hours, the country and the dose using a Generalized Estimating Equation model. Survival curves of the worms were explored in a univariate analysis (Kaplan-Meier curves). The median values of survival times were given. The survival curves were compared using log-rank test. A multivariate survival analysis was then performed (Cox model). No procedure of variables selection was performed. The assumptions of proportional hazards were checked. A value of p ≤ 0.05 given by the SAS^®^/ETS software (version 8.1) (SAS Institute Inc, Cary, NC, USA) was considered statistically significant.

## Results

### *In vitro *anti-adhesion activity of cranberry powder

Bacterial anti-adhesion activity of the whole Urell^®^/Ellura™ cranberry powder (measured by MRHA activity) was 0.47 mg/mL (data not shown).

### Effects detected by *ex vivo *assays

MRHA results indicated significant anti-adhesion activity (AAA) in urines collected from volunteers that consumed cranberry powder compared to placebo (p < 0.001) (Table 1). This inhibition was clearly dose-dependent, increasing with the amount of PAC equivalents consumed in each cranberry powder regimen. A peak of AAA was determined over the time course of the experiment. In urines samples collected at 6 h, there was a significant difference between the cranberry dosages containing 18 mg PAC and those with either 36 or 72 mg PAC (p = 0.002); however, no statistical difference was detected between 36 and 72 mg PAC. In urine samples collected at 24 h, there was a significant difference between the AAA of urines belonging to patients who had consumed cranberry dosages containing 72 mg of PAC (50% AAA) and the AAA of urines belonging to patients who had consumed 18 or 36 mg of PAC (0 and 12% AAA, respectively) (p = 0.002) (Table 1). In each country, similar results and trends were detected (Table 1).

**Table 1:** Urinary bacterial Anti-Adhesion Activity (AAA) detected with Mannose-resistant Hemagglutination (MRHA) assay (A) and urinary bacterial adhesion to T24 cells (B) expressed as Adhesion Index (AI) following consumption of increasing doses of cranberry powder standardized for 18, 36 or 72 mg of proanthocyanidins (PAC) vs placebo by participants in the 4 countries.

A	AAA				P			
					
Regimen	Placebo	18 mg PAC	36 mg PAC	72 mg PAC	18 vs 36	36 vs 72	6 h vs 24 h	Sp-Fr vs Ja-Hu
**Spain (n = 8)**								
**0 h**	0% [0-0]	0% [0-0]	0% [0-0]	ND				
**1-6 h**	0% [0-0]	50% [50-100]	100% [50-100]	ND	0.002	ND	<0.001	<0.001
**24 h**	0% [0-0]	0% [0-0]	12.5% [0-50]	ND	0.01	ND	<0.001	
	
**France (n = 8)**								
**0 h**	0% [0-0]	0% [0-0]	0% [0-0]	ND				
**1-6 h**	0% [0-0]	50% [50-100]	75% [50-100]	ND	0.005	ND	<0.001	
**24 h**	0% [0-0]	0% [0-0]	0% [0-0]	ND	NS	ND	<0.001	
	
**Japan (n = 8)**								<0.001
**0 h**	12.5% [0-50]	ND	25% [0-50]	25% [0-50]				
**1-6 h**	0% [0-0]	ND	100% [100-100]	100% [100-100]	ND	NS	<0.001	
**24 h**	0% [0-0]	ND	12.5% [0-50]	75% [50-100]	ND	0.001	0.005	
	
**Hungary (n = 8)**								
**0 h**	0% [0-0]	ND	0% [0-0]	0% [0-0]				
**1-6 h**	0% [0-0]	ND	87.5% [50-100]	100% [100-100]	ND	NS	<0.001	
**24 h**	0% [0-0]	ND	25% [0-50]	25% [0-50]	ND	NS	<0.001	
	
**Total (n = 32)**								
**0 h**	0.3% [0-0]	0% [0-0]	0.6% [0-50]	12.5% [0-50]				
**1-6 h**	0% [0-0]	50% [50-100]	90.6% [50-100]	100% [100-100]	0.003	NS	<0.001	ND
**24 h**	0% [0-0]	0% [0-0]	12.5% [0-50]	50% [50-100]	0.01	<0.001	<0.001	ND

**B**	**AI Median [Range]**				**P**			
					
**Regimen**	**Placebo**	**18 mg PAC**	**36 mg PAC**	**72 mg PAC**	**18 vs 36**	**36 vs 72**	**6 h vs 24 h**	**Sp-Fr vs Ja-Hu**

**Spain (n = 8)**								
**1-6 h**	18.5 [15-22]	8.5 [5-14]	5.0 [1-11]	-	<0.001	ND	<0.001	<0.001
**24 h**	20.0 [18-22]	14.5 [10-18]	12.5 [5-17]	-	<0.001	ND	<0.001	
	
**France (n = 8)**								
**1-6 h**	17.5 [15-28]	8.0 [5-16]	4.0 [2-10]	-	<0.001	ND	<0.001	
**24 h**	19.5 [18-25]	17.5 [12-22]	5.0 [2-10]	-	<0.001	ND	<0.001	

**Japan (n = 8)**								<0.001
**1-6 h**	21.5 [18-28]	-	5.0 [1-9]	3.5 [1-6]	ND	NS	<0.001	
**24 h**	25.5 [17-29]	-	13.5 [10-18]	10.0 [2-15]	ND	0.01	<0.001	
	
**Hungary (n = 8)**								
**1-6 h**	18.5 [15-22]	-	2.0 [1-5]	1.0 [1-5]	ND	NS	<0.001	
**24 h**	20 [15-25]	-	9.5 [5-25]	4.5 [2-10]	ND	<0.001	<0.001	

**Total (n = 32)**								
**1-6 h**	21.5 [15-28]	10.5 [5-16]	5.9 [1-11]	2.5 [1-6]	<0.001	<0.001	<0.001	ND
**24 h**	22.3 [15-29]	16.0 [10-22]	14.5 [2-25]	9.9 [2-15]	<0.001	<0.001	<0.001	ND

The results are representative of at least three independent trials. NS, not significant; ND, not determined. 0 h, pre-cranberry consumption collection

**Figure 1:**
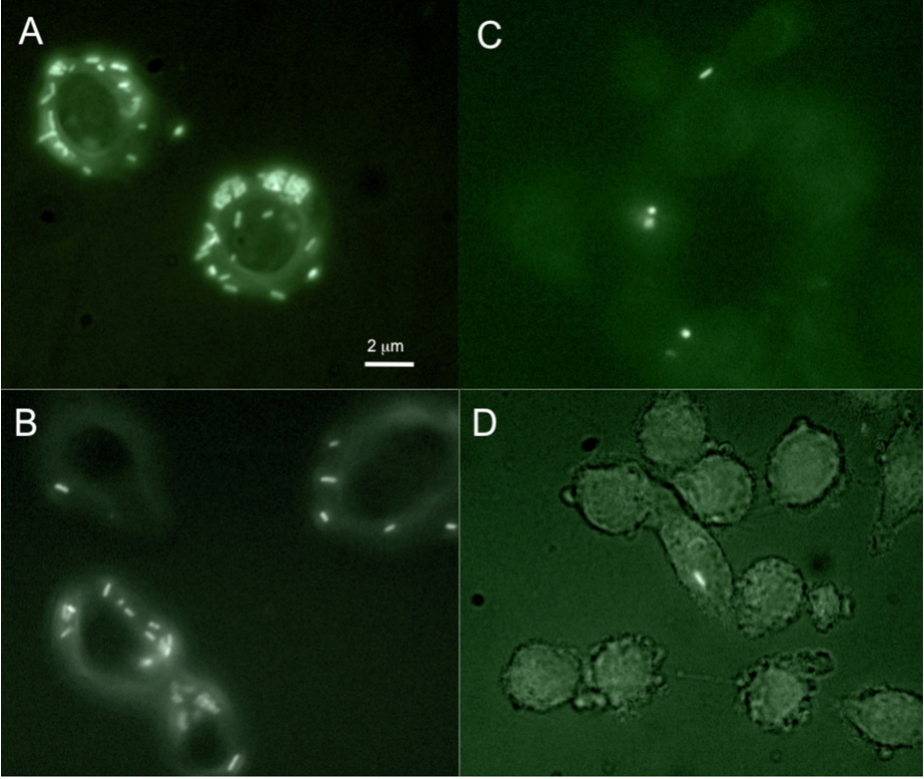
**Fluorescence microscopy of *E. coli gfp *+ strain cultured in urines of volunteers collected after cranberry powder consumption and loaded on T24 epithelial cells**. A. *E. coli *cultured in urines collected after placebo consumption; B. *E. coli *cultured in urines collected 6 h after consumption of cranberry powder containing 18 mg proanthocyanidins (PAC); C. *E. coli *cultured in urines collected 6 h after consumption of cranberry powder containing 36 mg PAC; D. *E. coli *cultured in urines collected 6 h after consumption of cranberry powder containing 72 mg PAC.

The *ex vivo *epithelial cell adhesion assay results indicated a highly significant reduction in bacterial adhesion to T24 cells compared to placebo (p < 0.001) following the consumption of cranberry dosages containing 36 or 72 mg of PAC (Table 1, Figure 1). There was a dose-dependent decrease in bacterial adhesion with cranberry intake. The Adhesion Index (AI) of bacteria grown in urine samples collected after consumption of cranberry with 36 or 72 mg PAC was significantly lower than the AI following the dose with 18 mg PAC (p < 0.001). The multivariate analysis clearly confirmed a dosage effect for each cranberry regimen vs placebo (p < 0.001) (Table 2). AI of bacteria in urines collected at 6 h and at 24 h were significantly different following all cranberry regimens (p < 0.001). Interestingly, the AI at 24 h after cranberry intake of 72 mg PAC equivalents remained significantly low (AI median <10) compared to the other regimens (p < 0.001). The multivariate analysis confirmed a time dependent-effect with the maximum effect at 6 h compared to 24 h (p < 0.001). The multivariate analysis demonstrated an effect of patients' origin. When the data were adjusted by hour and dose, results from Japan were significantly different compared to the three other countries, with higher AI (p < 0.001) (Table 2).

**Table 2:** Multivariate analysis of results obtained to urinary bacterial adhesion to T24 cells expressed as adhesion index.

		Parameters	SD	IC95%		p	p globale
						
				inf	sup		
***Countries***	***France***	.		.	.		<0.001
	***Spain***	-1.649	1.4478	-4.487	1.188	0.26	
	***Japan***	3.127	1.6099	-0.028	6.283	0.05	
	***Hungary***	0.332	1.7194	-3.038	3.702	0.85	

***Hours***	***1-6 H***	.	.	.	.		
	***24 H***	7.089	0.7367	5.645	8.533	<0.001	

***Dose***	***Placebo***	.	.	.	.		<0.001
	***36 mg***	-9.236	1.1482	-11.487	-6.986	<0.001	
	***72 mg***	-16.595	0.6769	-17.921	-15.268	<0.001	
	***144 mg***	-20.929	0.9071	-22.0707	-19.151	<0.001	

The index value was modelled by the hours, the country and the dose using a Generalized Estimating Equation model.

At 6 h for the 3 PAC equivalency doses, a significant correlation between AAA results and AI results was noted (p = 0.01, 0.04 and 0.04 for 18, 36 and 72 mg PAC). This trend was not observed at 24 h for the 3 doses (p = non significant, 0.94 and 0.10 for 18, 36 and 72 mg PAC).

### Reduction of *E. coli *virulence

We evaluated the virulence potential of *E. coli *strain in presence of different urines following consumption of cranberry powder. The mean survival time (LT50) for worms was significantly increased to 5-6 days with *E. coli *grown in urine samples collected after cranberry intake compared to LT50 for strain grown in urine samples collected after placebo intake (3 days) (p < 0.001) (Table 3). In urines collected after 6 h, no difference can be detected between the virulence of *E. coli *strain grown in urines collected after consumption of PAC.

**Table 3:** *vivo *kinetics of killing of *C. elegans *infected by *E. coli *grown in urine samples after consumption of the different proanthocyanidins regimens.

A	LT50 Median (IC95%) in days				P				
**Regimen**	**Placebo**	**18 mg**	**36 mg**	**72 mg**	**Pl vs 18/36**	**18 vs 36**	**36 vs 72**	**6 h vs 24 h**	**Sp-Fr vs Ja-Hu**

**Spain**									

**1-6 h**	3.5 [3-5]	5 [3-7]	6 [5-8]	ND	<0.001	<0.001	ND	NS except for 72 mg	
	
**24 h**	3 [3-6]	4.5 [3-6]	5 [3-6]	ND	<0.001	NS	ND	<0.001	
	
**France**									<0.001
	
**1-6 h**	3 [3-5]	5 [4-7]	5.5 [4-7]	ND	<0.001	NS	ND	NS except for 36 mg	
	
**24 h**	3 [3-5]	3 [2-4]	5 [4-8]	ND	NS	<0.001	ND	<0.001	

**Japan**									
	
**1-6 h**	4 [3-5]	ND	6.5 [5-9]	7 [5-9]	<0.001	ND	NS	NS	
	
**24 h**	4 [3-5]	ND	5.5 [4-8]	6 [4-8]	<0.001	ND	NS		
									<0.001
**Hungary**									
	
**1-6 h**	3 [3-5]	ND	5 [4-8]	6 [5-10]	<0.001	ND	NS	NS	
	
**24 h**	4 [3-5]	ND	5 [4-8]	6 [4-9]	<0.001	ND	NS		

**Total**									

**1-6 h**	3 [3-5]	5 [3-7]	5.5 [4-9]	6.5 [5-10]	<0.001	NS	<0.001	NS	ND

**24 h**	3.5 [3-5]	4 [2-6]	5 [3-8]	6 [4-9]	<0.001	<0.001	<0.001		ND

**OP50**	6 [6-7]								

**B**	**LT100 Median (IC95%) in days**	**P**							

**Regimen**	**Placebo**	**18 mg**	**36 mg**	**72 mg**	**Pl vs 18/36**	**18 vs 36**	**36 vs 72**	**6 h vs 24 h**	**Sp-Fr vs Ja-Hu**

**Spain**									

**1-6 h**	8.5 [7-8]	10 [9-10]	11.5 [11-12]	ND	<0.001	<0.001	ND	NS except for 72 mg	<0.001

**24 h**	7 [6-8]	9 [8-10]	9.5 [9-10]	ND	<0.001	NS	ND	<0.001	

**France**									

**1-6 h**	7 [6-8]	10 [9-11]	11 [10-12]	ND	<0.001	NS	ND	NS except for 36 mg	

**24 h**	7 [6-8]	8 [7-9]	10.5 [10-11]	ND	NS	<0.001	ND	<0.001	

**Japan**									<0.001

**1-6 h**	8 [7-8]	ND	12.5 [12-13]	13 [12-14]	<0.001	ND	NS	NS	

**24 h**	7.5 [7-8]	ND	11 [10-12]	12 [11-13]	<0.001	ND	NS		

**Hungary**									

**1-6 h**	7 [6-8]	ND	12 [11-13]	12.5 [12-13]	<0.001	ND	NS	NS	

**24 h**	8 [7-8]	ND	11 [10-12]	11.5 [11-12]	<0.001	ND	NS		

**Total**									

**1-6 h**	7 [6-8]	5 [3-7]	5.5 [4-9]	6.5 [5-10]	<0.001	NS	<0.001	NS	ND

**24 h**	8 [7-8]	4 [2-6]	5 [3-8]	6 [4-9]	<0.001	<0.001	<0.001		ND

**OP50**	14 [13-15]								

(A) Determination of LT50 Median; (B) Determination of LT100 Median.

In multivariate analysis, there was a significant effect of PAC dose influencing the virulence of *E. coli *(HR = 1 for placebo to HR = 0.32 for 72 mg): there was an inverse relationship between consumed PAC and the virulence of *E. coli *observed (P < 0.001) (Table 2). So, the effect of PAC was "protective" for the worms. Moreover there was an effect of hour on the virulence of *E. coli*. The virulence of *E. coli *against worms was increased to 32% with urines collected at 24 h compared to urines collected at 6 h (P < 0.001).

The number of *E. coli *CFU within the nematode gut varied around 10^6 ^bacteria per worm for each strain 72 h after ingestion without statistical difference (data not shown) confirming the ingestion and proliferation of *E. coli *isolates in the *C. elegans *intestine.

## Discussion

In this study, two experimental assays were optimized successfully to demonstrate bacterial anti-adhesion activity in human urines following cranberry powder consumption. Two collection periods were chosen: 1-6 h corresponding to the most important elimination period of PAC in urines as previously demonstrated by Howell et al. [11] and 24 h, the residual period of PAC elimination in urines. Results for the MRHA assay were inversely correlated with the epithelial cell adhesion assay for the first urines collected from 1-6 h after ingestion of the various dosages of cranberry PAC equivalents (*e.g. *AI decreased when anti-adhesion activity determined by MRHA increased). However this correlation was not noted at 24 h, implying that the epithelial cell adhesion assay may be more sensitive to detecting activity in urines when the active metabolites are less concentrated. Our results suggest that both the MRHA and the epithelial cell adhesion assays could potentially be utilized to screen for urinary anti-adhesion activity, especially in the first 6 h following cranberry ingestion.

Adaptation of the cell adhesion assay using a fluorescent *E. coli *strain eliminates false positives observed in previous experiments using Giemsa to stain bacteria [12,13]. In the experimental protocol used in the present study, the analysis is faster and easier than with Giemsa, providing an improvement in quantification of bacterial adhesion.

This multi-location randomized, double-blind versus placebo study revealed a dose-dependent and time-dependent effect in two different *ex vivo *models of *E. coli *adherence to bladder epithelial cells. We demonstrated a peak of urinary anti-adhesion activity 6 h after ingestion of the PAC-standardized cranberry powder, with a reduction in activity at 24 h. There was a linear increase in urinary anti-adhesion activity with increasing dosages of PAC equivalents at both 6 h and 24 h. Interestingly a residual anti-adhesion effect was shown 24 h after consumption of 36 mg of PAC equivalents, with a more profound effect after 72 mg. This effect was evident in urines from all volunteers, regardless of country location. These results highlighted for the first time that to achieve a bacterial anti-adhesion effect in urine, 36 mg of cranberry PAC equivalents per day is effective, but 72 mg may offer a nyctohemeral protection. The kinetic data indicate that activity is highest after 6 h for both 36 and 72 mg, but decreases significantly after 24 h, suggesting that it may be beneficial to consume cranberry in two split doses of 36 mg in the morning and evening. With 72 mg of PAC, the different metabolites of PAC (notably different anthocyanins [27]) are always present and efficient 24 h after cranberry consumption. Further human trials are needed to correlate the level of *ex vivo *anti-adhesion activity with prevention of clinical UTI.

The pre-study background urines of several of the Japanese volunteers exhibited anti-adhesion activity. This phenomenon could be due to the production of endogenous adhesion inhibitors (such as Tamm-Horsfall glycoprotein) that are produced by some people. These inhibitors are known to be transitory and can be induced by a number of factors, dietary (on salt and water balance) and environmental. However, the AI in this population was significantly higher compared to other populations in multivariate analysis (p < 0.001). As the influence of dosage and hour was suppressed in this analysis, this difference could be explained by the effect of diets between the Asian and European lifestyles.

The PAC contained in cranberry inhibit *E. coli *adhesins notably against P-fimbriae [3,7]. Adhesion is the first step of bacterial virulence, to ensure the survival and establish its replication. To demonstrate the effect of PAC on bacterial virulence, we used an *in vivo *killing nematode model validated by different teams [12,25,26,28,29]. We determined that the reduced ability of *E. coli *strain to kill worms correlates with the consumption of cranberry capsules. Bacteria grown in the urine of individuals consuming the cranberry capsule are not able to adhere to the worms and thus exhibit reduced killing of the worms in spite of the presence and proliferation of *E. coli *strain in the *C. elegans *intestine. *E. coli *were localized within the nematode gut but could not exert any virulent effect because they can establish any infection and loss of virulence.

## Conclusions

Administration of PAC-standardized cranberry powder at dosages containing 72 mg of PAC per day may offer a nyctohemeral protection against bacterial adhesion and virulence in the urinary tract. Since bacterial adhesion is the primary step in initiation of UTI, consumption of cranberry may offer an additional means to help prevent infections.

## Abbreviations

AAA: Anti-Adhesion Activity; AI: Adhesion Index; MRHA: mannose-resistant hemagglutination; PAC: proanthocyanidins; UTI: urinary tract infection

## Competing interests

HB and TM have participated in trials organized by Pharmatoka. ABH, CC, ABBP, LG, PT, AS and JPL have declared that no competing interests exist. Pharmatoka had no role in the study design, data collection and analysis, decision to publish or preparation of the manuscript.

## Authors' contributions

ABH, JPL and AS conceived of the study, participated in its design and coordination, carried out the assays and drafted the manuscript. HB participated in the design of the study and coordination, enrolled the volunteers, and helped to draft the manuscript. CC performed statistical analysis and helped to draft the manuscript. ABBP carried out the assays on urothelial cells and helped to draft the manuscript. LG, TM, PT enrolled the volunteers in each country, followed the study and helped to draft the manuscript. All authors read and approved the final manuscript.

## Pre-publication history

The pre-publication history for this paper can be accessed here:

http://www.biomedcentral.com/1471-2334/10/94/prepub
